# Enzyme-Responsive Nanoparticles for Anti-tumor Drug Delivery

**DOI:** 10.3389/fchem.2020.00647

**Published:** 2020-07-30

**Authors:** Mengqian Li, Guangkuo Zhao, Wei-Ke Su, Qi Shuai

**Affiliations:** Collaborative Innovation Center of Yangtze River Delta Region Green Pharmaceuticals, Zhejiang University of Technology, Hangzhou, China

**Keywords:** enzyme-responsive, nanomedicine, stimuli-responsive, controlled release, cancer

## Abstract

The past few decades have seen great progress in the exploration of nanoparticles (NPs) as novel tools for cancer treatments and diagnosis. Practical and reliable application of nanoparticle-based technology in clinical transformation remains nevertheless an ongoing challenge. The design, preparation, and evaluation of various smart NPs with specific physicochemical responses in tumor-related physiological conditions have been of great interests in both academic and clinical research. Of particular, smart enzyme-responsive nanoparticles can predictively and selectively react with specific enzymes expressed in tumor tissues, leading to targeted delivery of anti-tumor drugs, reduced systemic toxicity, and improved therapeutic effect. In addition, NPs interact with internal enzymes usually under mild conditions (low temperature, aqueous media, neutral or close to neutral pH) with high efficiency. In this review, recent advances in the past 5 years in enzyme-responsive nanoparticles for anti-tumor drug delivery are summarized and discussed. The following contents are divided based on the different action sites of enzymes toward NPs, notably hydrophobic core, cleavable/uncleavable linker, hydrophilic crown, and targeting ligand. Enzyme-engaged destruction of any component of these delicate nanoparticle structures could result in either targeting drug delivery or controlled drug release.

## Introduction

Cancer is one of the leading threats to human health and one of the main causes of death worldwide (Siegel et al., 2020). Traditionally, chemotherapy has been given high priority to treat cancer due to its great potential to cure early-stage cancers, as well as its possibility to improve the life quality of patients with advanced cancers. However, conventional chemotherapeutic agents are normally distributed non-specifically in the body and both cancerous and normal cells are affected, leading to serious side effects and compromised therapeutic effects. It is true that the lack of this specificity could be overcome by developing molecular targeted drugs (Ross et al., 2004) but the rapid development of drug resistance during the treatment is still a tough nut (Morgillo and Lee, 2005). In the past few decades, cancer nanotherapeutics have been undergoing rapid development. Among them, nanoparticles (NPs), as novel drug delivery carrier, have been extensively studied to solve the limitations of conventional chemotherapeutics, such as non-specific biodistribution, poor water solubility, low therapeutic indices, and proneness to drug resistance (Cho et al., 2008). Several therapeutic NPs have been successfully developed and launched on market, including Abraxane® and Doxil® which were, respectively approved for the treatment of metastatic breast cancer and pancreatic cancer (Poveda et al., 2005). In addition, Ontak®, Onivyde®, and DepoCyt® have also been approved by FDA for clinical use (Ventola, 2017), which indicates the bright marketing prospect of therapeutic NPs.

**Figure 1:**
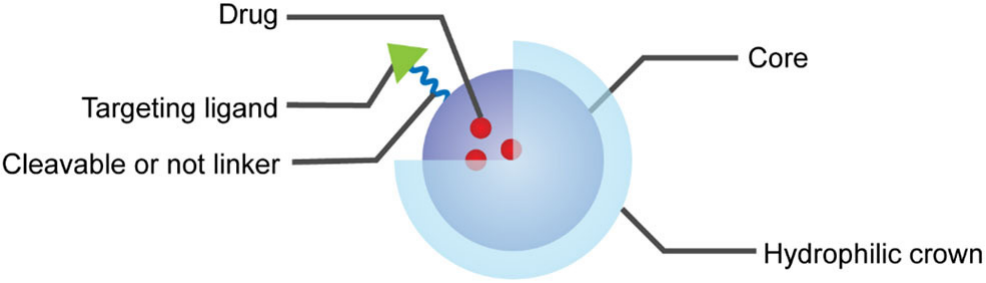
Multifunctional and drug-loaded nanoparticles contain part or all of these components: core (usually loaded with drug), linker, hydrophilic crown, and targeting ligand.

Various types of carriers have been used in cancer nanotherapeutics, including liposomes, polymeric NPs and micelles, metallic NPs, carbon nanotubes, solid lipid NPs, niosomes, and dendrimers (Torchilin, 2014). A wide variety of payloads, such as small molecular drugs, proteins, peptides, nucleic acids, vaccines, antibody, and so on, can be loaded and delivered through physical encapsulation, covalent conjugation, surface attachment or interception (Hans and Lowman, 2002). Diverse formulations based on versatile NPs have been successfully explored to deliver drugs to lymphatic system, brain, arterial wall, lung, liver, spleen, and other organs with long-term circulation and controlled release profile (Hans and Lowman, 2002). Usually, the size of formulated NPs ranges from a few nanometers to several 100 nm, which enables the NPs with passive targeting ability and achieves desired enrichment of payloads in tumor tissues through the enhanced permeability and retention (EPR) effect (Haley and Frenkel, 2008). In addition, with the attachment of targeting ligands or antibodies on the surface of NPs, they are potentially endowed with positive targeting ability (Kamaly et al., 2012). Both of these passive targeting ability and positive targeting ability of NPs are of great importance in reducing the side effects of anti-tumor drugs loaded and improving their therapeutic efficacy. Depending on the specific properties of drugs loaded and the desired delivery pathways, a few strategies for the design and preparation of NPs have been presented. In general, the typical structure of multifunctional, drug-loaded NPs can be roughly illustrated as follows: core (usually hydrophobic), cleavable or not linker, hydrophilic crown, and targeting ligand, as illustrated in Figure 1.

## Multifunctional and Stimuli-Sensitive Nanoparticulate Drug Delivery System

**Figure 2:**
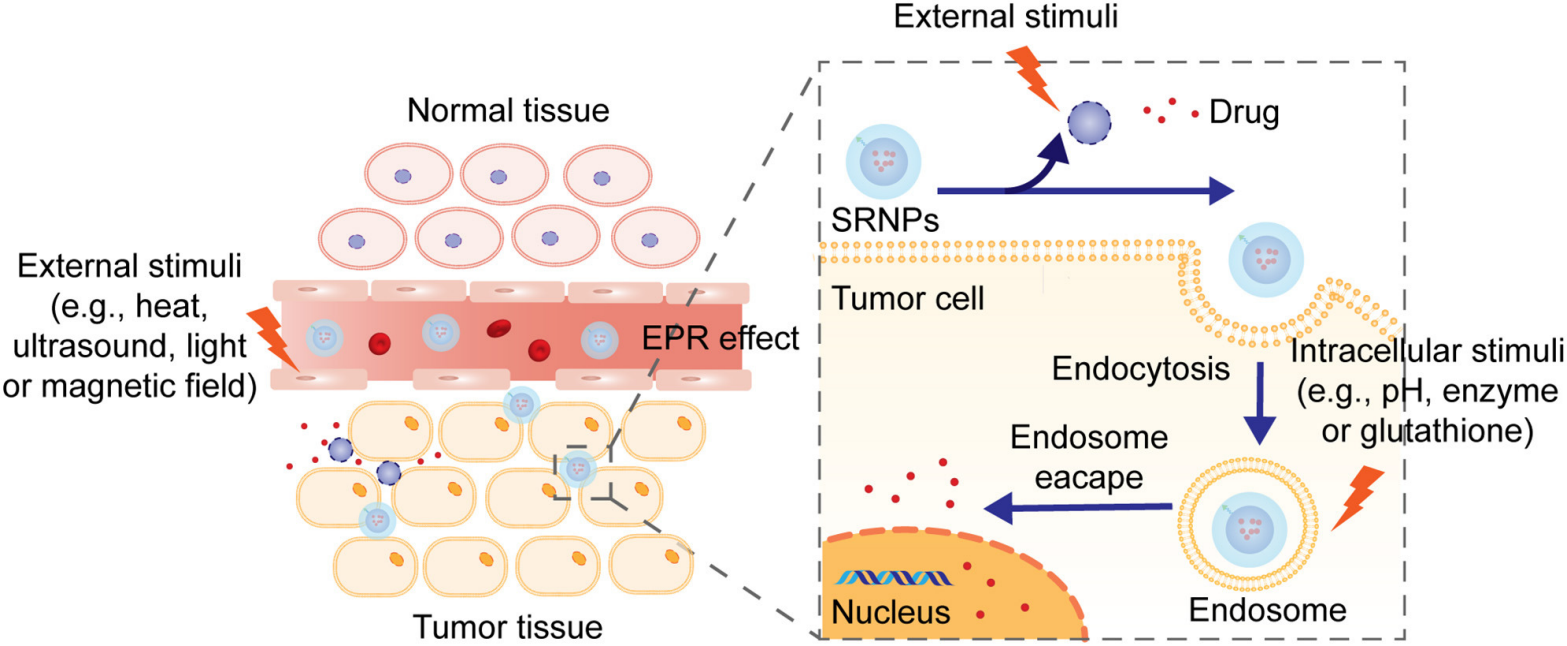
The mechanism of drug release from stimuli-responsive nanoparticles. Nanoparticles penetrate into the tumor tissues through EFR effect or with the assistance of targeting ligands. External or internal stimuli induce structural collapsion of the nanoparticles, which prompts the release of drug at target location.

Different from traditional NPs, stimuli-responsive nanoparticles (SRNPs) have been considered as promising carriers because of their unique bio-responsive physicochemical characteristics and numerous successful applications of SRNPs have been demonstrated. These “smart” SRNPs can react in a predictable and specific way to external or internal stimuli (Karimi et al., 2016), as illustrated in Figure 2. In response to a range of endogenous stimuli, such as changes in pH (Lee et al., 2005; Ko et al., 2007, 2010; Lee E. S. et al., 2008; Min et al., 2010; Yang G. B. et al., 2018), hypoxia (Lin et al., 2013; Lee et al., 2017; Ihsanullah et al., 2020), enzyme-specific expression (Lee S. et al., 2008; Lee et al., 2009; Choi et al., 2011, 2014; Zhao et al., 2019), redox state (Li et al., 2012; Shi et al., 2014; Xu et al., 2018), reactive oxygen species (Kim et al., 2014; Deepagan et al., 2016; Yang Z. et al., 2018) in diseased tissues or intracellular compartments, SRNPs undergo changes in molecular structure, solubility, and surface properties, shape and self-association or dissociation behaviors, which can facilitate cellular uptake, improve endosomal escape or trigger either intracellular or extracellular drug release. In addition, SRNPs can also respond to exogenous stimuli, such as laser irradiation (Han et al., 2016) and temperature changes (Kono, 2001; Li et al., 2013; Limmer et al., 2014), to generate an off/on activation of imaging or therapeutic function. Furthermore, well-designed smart nanoparticles can even respond to combinations of multiple stimuli to further improve their specificity for targeted drug delivery and controlled drug release (Cheng et al., 2013; Chen et al., 2020; Hou et al., 2020; Yu et al., 2020). This specificity allows nanoparticles to release their payload precisely in a temporal or spatial pattern in response to specific pathological triggers present in the diseased tissues, which is supposed to reduce side effects, achieve dosing on demand, and increase therapeutic efficacy (Mura et al., 2013).

Among them, enzyme-responsive nanoparticles have been considered as one of the most promising smart stimulus-responsive nanoparticles. First of all, changes in the expression of specific enzymes, such as proteases, phosphatases, and glycosidases, can be observed in tumor or inflammatory regions, which can be exploited to achieve targeted accumulation of drugs at the desired biological location via enzyme-mediated drug release (Mura et al., 2013). For example, it is reported that the expression level of prostate-specific membrane antigen (PSMA, also known as glutamate carboxypeptidase 2) in prostatic cancer cells is 100-fold to 1000-fold to normal prostate epithelial cells (Troyer et al., 1995; Silver et al., 1997; Bostwick et al., 1998; Mannweiler et al., 2009; Maurer et al., 2015). Cathepsin B is overexpressed in various types of cancers including breast, lung, prostate, colorectum, and endometrium (Aggarwal and Sloane, 2014). Moreover, enzymes, as triggers, have many advantages, including high chemical selectivity and substrate specificity (de la Rica et al., 2012), and usually enzyme-catalyzed reactions proceed efficiently under mild conditions (low temperature, aqueous media, neutral or close to neutral pH) (Ulijn, 2006; Hu et al., 2012). For example, phospholipase A2 (sPLA2) can degrade the fatty ester group at the sn-2 position of glycerophospholipids with extremely high selectivity (Dennis et al., 2011). Plasmin can preferentially catalyze the hydrolysis of peptide bonds formed by arginine or lysine (West and Hubbell, 1999; van Dijk et al., 2010). Only at neutral pH, Cathepsin B can act as an endopeptidase and catalyze the hydrolysis of large peptide substrates (Aggarwal and Sloane, 2014). Active tumor-targeting nanoparticles integrated with site-specific enzyme-triggered moieties are able to significantly achieve enhanced accumulation at the tumor site, reduced undesired uptake by non-targeted tissues, as well as site-specific controlled drug release (Allen, 2002).

In this review, we will focus on significant progress in the field of enzyme-responsive nanoparticles for anti-tumor drug delivery in the past 5 years. We will initially introduce the general mechanism for controlling enzyme-responsive drug release from nanoparticles. And then, key examples of drug delivery and disease diagnosis systems based on enzyme-responsive nanoparticles will be presented, which will be organized based on different installation sites of specific enzyme bioactive functionalities on nanoparticles. Critical discussion and an outlook for these systems will also be provided.

## General Mechanism for Enzyme-Responsive Controlled Drug Release From NPs

In human body, every biological and metabolic process seriously relies on the actions of enzymes. Drug release from NPs in an enzyme-responsive way is origin from the specific enzyme-catalyzed chemical reactions which lead to degradation, dissociation, or morphological transitions of the parent NPs (Torchilin, 2014). In order to achieve controlled release profile of drugs, severe degradation of NPs exposed to enzymes, which usually leads to burst release of drugs, is neither necessary nor preferred. In tumor microenvironment with the presence of specific enzymes, controlled changes in macro-scale structure of NPs usually afford desired controlled release of drugs (Kamaly et al., 2016; Wang et al., 2019).

As we mentioned above, the delicate structure of NPs is normally consisted of four components and decomposition of any component can potentially result in the destruction of integrity of NPs, followed by the release of drugs encapsulated. This lies in the premise for the design of enzyme-responsive nanoparticles and any component is possible to be attached with enzyme-sensitive moiety which is usually a substrate or a substrate mimic of the enzyme. In addition, a second component is responsible for changes in the internal interactions, which can eventually lead to macroscopic transitions and drug release from NPs (Karimi et al., 2016). Principally, depending on the drug delivery and release demand, the action site of enzyme can be located on any component of NPs carriers, as long as it bears enzyme-sensitive functionality. Therefore, the examples in this review will be divided into the following four patterns and discussed in detail.

## Enzyme-Responsive Nanoparticles

### Nanoparticles With Enzyme-Responsive Core

Within the core of NPs are located the active drugs, which are entrapped via physical interactions or chemical covalent conjugation. Upon the action of enzymes toward functionality installed in the core, the release of drugs can be triggered by changes in structure, such as disintegration, macroscopic deformation, charge switching, breakage of covalent bonds, and so on (Zhou et al., 2019).

**Figure 3:**
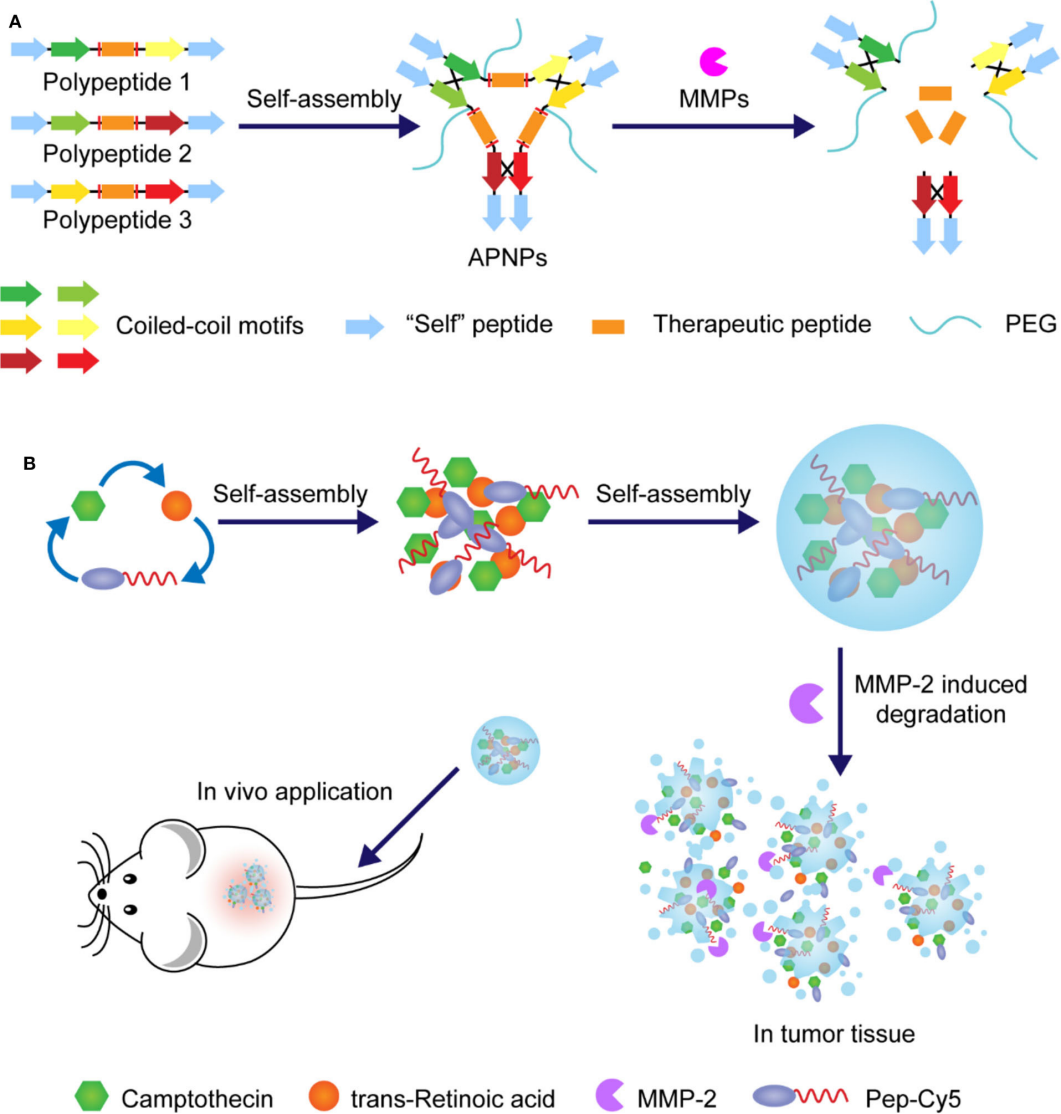
**(A)** Schematic illustration and action mechanism of APNPs which were self-assembled from protease-sensitive peptides. Therapeutic peptide was released with the hydrolysis of substrate peptides. **(B)** Schematic illustration of preparation of nanoparticles through assembly of amphipathic Pep-Cy5 with two hydrophobic drugs and disintegration under the action of MMP-2.

One of the most common methods of preparing nanoparticles with enzyme-responsive core is by self-assembly of peptides with enzyme-cleavable sequences or covalent conjugation of proteinase-sensitive peptides to therapeutic or diagnostic agents. In this case, matrix metalloproteinases (MMPs), a family of over 20 calcium-dependent zinc-containing proteinases, have been demonstrated the ability to catalyze the core degradation of peptide-based NPs. Among them, MMP-2 and MMP-9 are of particular importance for the development of enzyme-responsive anti-tumor drug delivery systems due to their proved correlation with cancer cell invasion and metastasis formation (Egeblad and Werb, 2002; Yoon et al., 2003). For example, Zhou and coworkers developed novel enzyme-responsive activatable protein nanoparticles (APNPs) for the targeting delivery of therapeutic peptides (Yu et al., 2018). In this study, the core of these PEG-coated APNPs was constructed by self-assembly of peptides embedded with therapeutic peptides and activatable toward proteases with high expression levels in the microenvironment of diseased tissues, as illustrated in Figure 3A. It was designed to achieve extended circulation time *in vivo*, reduced systemic toxicity, targeted delivery, and controlled release of therapeutic peptides. Melittin (Mel), a promising anticancer agent as cargo, was used to evaluate this delivery platform, in which the peptides were specifically engineered with MMP-responsive PLGLAG sequences. It was demonstrated that, compared with free melittin, these finely tuned Mel-APNPs exhibited limited toxicity. However, desired comparable cytotoxicity was observed after exposure to MMP-2 due to the enzyme-triggered release of active melittin from Mel-APNPs. These delicate Mel-APNPs were further upgraded to TfR-Mel-APNPs with targeting ability. Targeted delivery and controlled release of melittin *in vivo* were successfully achieved, making clinical transformation of these therapeutic peptides possible.

Similarly, in one case, the MMP-2 cleavable peptide sequence GPLGVRGE was attached to hydrophobic near-infrared dye Cy5, affording an amphiphilic multifunctional molecule Pep-Cy5 (Yang et al., 2016). Through self-assembling, this belt-shaped amphiphilic molecule would form water-soluble nanoparticles with hydrophobic anti-tumor drugs that could be a combination of different drugs, making it potential for synergistic administration, as illustrated in Figure 3B. In this study, anti-tumor drugs camptothecin and trans-retinoic acid were cooperatively entrapped in enzyme-responsive NPs, which exhibited a desired MMP-2-triggered degradation process and achieved reduced side effects, enhanced intertumoral accumulation, and improved anti-tumor efficacy.

**Figure 4:**
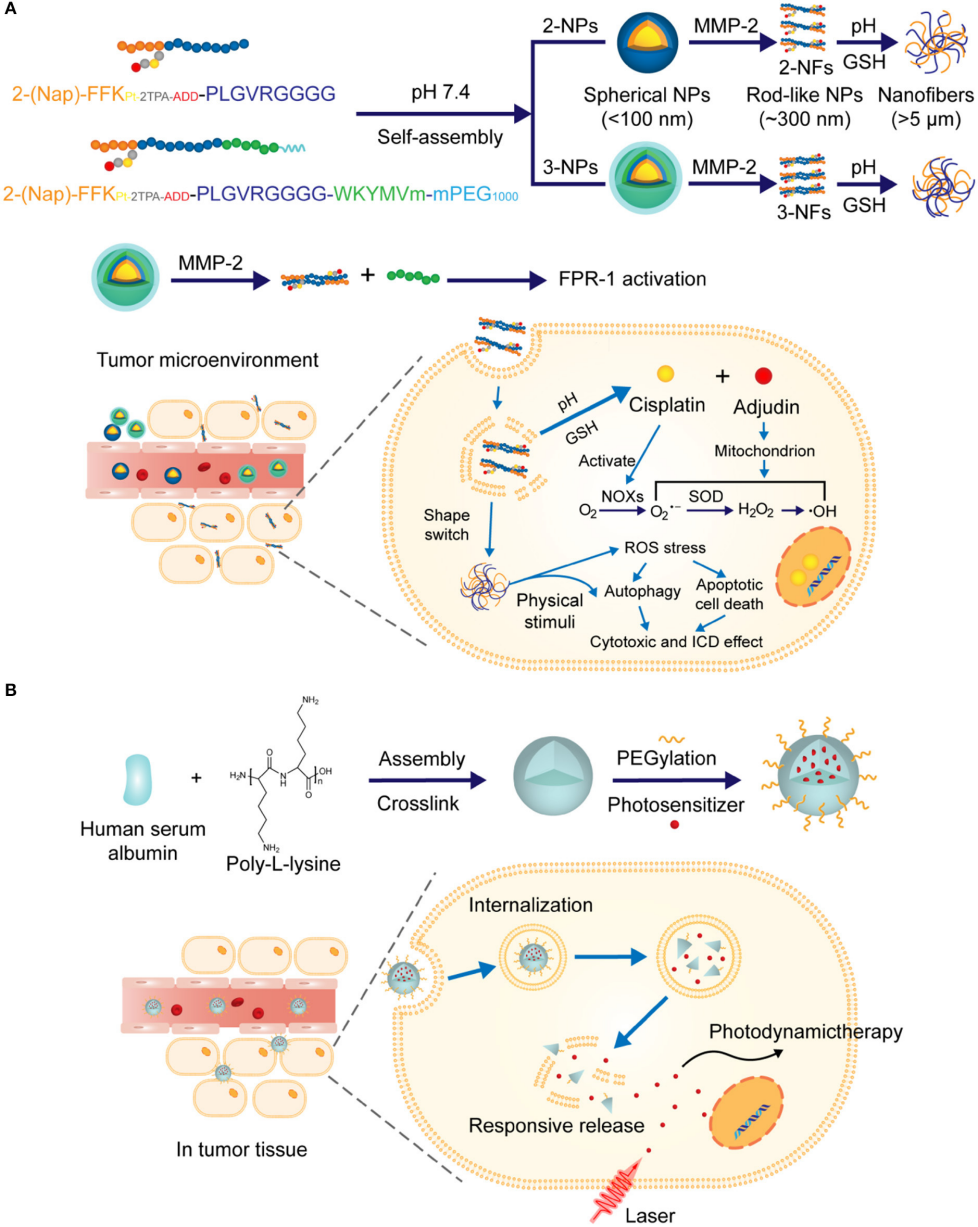
**(A)** Schematic illustration of the construction of nanoparticles capable of structural transformation activated by MMP-2 in tumor microenvironment. Rod-shaped particles were uptaken by the cell and drugs were released under the trigger of pH change and GSH. **(B)** Schematic illustration of construction of the nanoparticles via electrostatic assembly from proteinase substrate human serum albumin and poly-L-lysine. Photosensitizer was released under combined multiple triggers including proteinase K.

In another case, Zhang et al. reported an immunotherapy strategy for triple-negative breast cancer based on enzyme-responsive and structure-transformable nanoparticles, as illustrated in Figure 4A (Xu et al., 2019). Firstly, peptides containing anti-tumor agents cisplatin (Pt) and adjudin (ADD) and MMP-2-recognizable sequences were synthesized and then self-assembled into spherical nanoparticles with diameters less than 100 nm. It was demonstrated that after accumulation in the tumor bed with overexpressed MMP-2, these spherical nanoparticles underwent structural transformation into rod-like nanoparticles with prolonged drug retention time and deep tumor penetration capability. In addition, by adding WKYMV (a kind of FPR-1 agonist), further development of MMP-2-responsive NPs with three active components and additional hydrophilic PEG chains was realized, and similar structural changes from sphere to rod were observed. Remarkably, this enhanced version of nano-platform exhibited further improved antitumor immunity by synergistic activation and promotion of immunogenic cell death.

Like MMP, overexpression of proteinase for albumin catabolism in tumors has also been approved, which can be utilized to design proteinase-responsive NPs for antitumor drug delivery. For example, Zhang N. et al. (2016) developed a proteinase K involved multi-triggered nanoparticles based on human serum albumin, which was successfully applied for photodynamic tumor ablation. In this study, human serum albumin was used with synthetic polypeptide poly-L-lysine to prepare nanoparticles via electrostatic assembly and PEG was attached onto the surface of NPs, as illustrated in Figure 4B. The *in vitro* triggered release profile of photosensitizer Chlorin e6 from these NPs was evaluated in PBS solutions, which indicated that, compared with other stimuli, proteinase K significantly promoted the release of Chlorin e6 due to the accelerated degradation of NPs. Interestingly, the presence of combined multiple triggers including pH, glutathione (GSH) and proteinase K exhibited the fast release rate of Chlorin e6.

As a number of strategies have emerged to explore enzymes closely associated with specific diseases for biomedical applications, together with the high intrinsic complexity of enzyme-responsive NPs and subtle interactions between these delivery systems and diseased cells, it is quite necessary to establish a general mode for the rational design of enzyme-responsive delivery systems. As we mentioned above, MMPs are among those enzymes with top interest to researchers. Especially, MMP-9 was drawn from a cross-section of MMPs by Ulijn (2006), who presented guidance for the design of a customizable peptide-based NPs with excellent therapeutic effects (Son et al., 2019). They started with the design of peptide amphiphiles with ionic hydrophilic section, MMP-9-cleavable section and hydrophobic section. And then, they systemically studied the compatibility of the cleavable section with the entire nanoparticle system, the susceptibility of the nanoparticle to the scissor MMP-2, and the relationships between the morphology of the nanoparticle pre- and post-cleavage and its pharmacodynamic effects. Eventually, they demonstrated that surface charge, supramolecular organization and enzyme specificity of peptide-based nanoparticles could be customized by switching a few amino acids in the peptide sequences.

### Nanoparticles With Enzyme-Responsive Crown

Surface modification of nanoparticles with hydrophilic moieties is usually essential for its applications in drug delivery, in order to increase water-solubility, prevent drug leakage, avoid reorganization by the reticuloendothelial system (RES), improve interactions with cells, and facilitate cellular uptake. A wide range of materials with high hydrophilicity have been investigated, among which proteins or peptides, hyaluronic acid (HA), and synthetic polymers cross-linked by peptides are of great interest for the development of NPs with enzyme-responsive crown. Ideally, this hydrophilic auxiliary of NPs is supposed to slip off after the NPs reach the targeting action sites and facilitate the release of active drugs encapsulated. In this way, it will be highly desired for the development of enzyme-triggered deshielding approaches due to the close association of enzymes with specific diseases, especially tumors.

**Figure 5:**
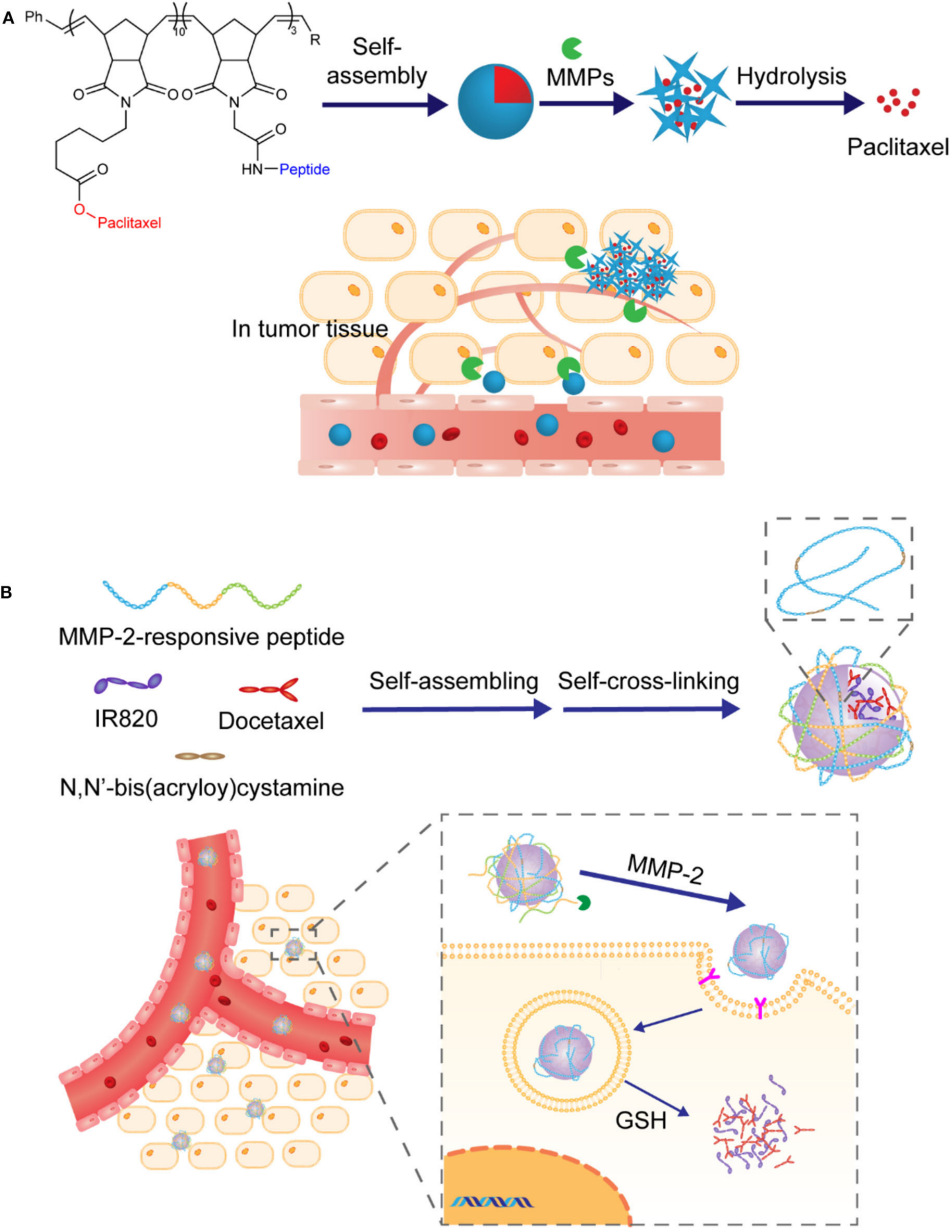
**(A)** Schematic illustration of nanoparticles prepared via self-assembling of amphiphilic block copolymers and drug release in response to MMPs. **(B)** Schematic illustration of the preparation of hybrid nanoparticles via assembly and self-cross-linking formation. Drugs were released in response to MMP-2 and GSH-triggered degradation of crown.

Enzyme-cleavable peptides are of high priority for this consideration. For example, Callmann et al. (2015) successfully constructed amphiphilic block copolymers via ring-opening polymerization of norbornene analogs, which were functionalized with hydrophobic paclitaxel and hydrophilic MMP-responsive peptide (GPLGLAGGERDG) via biodegradable ester bonds and amide bonds, respectively. The resulting amphiphilic block copolymers assembled into micellar nanoparticles coated with hydrophilic peptides which were cleaved upon exposure to MMP presented in the diseased tissue, as illustrated in Figure 5A. As a result, the open hydrophobic core turned accessible to hydrolysis and the active paclitaxel was effectively released, leading to enhanced accumulation of drugs and improved therapeutic efficacy. Moreover, in another case, the peptide-based crown was further stabilized by forming a cross-linking network structure without interference of its enzyme-sensitivity (Peng et al., 2019). In this study, the peptides with MMP-2-cleavable sequence in the middle were cross-linked by *N, N*′-bis (acryloyl) cystamine, as shown in Figure 5B. This was proved to be crucial for high drug loading capacity of these NPs and enhanced penetration due to the presence of additional GSH-responsive disulfide bonds.

**Figure 6:**
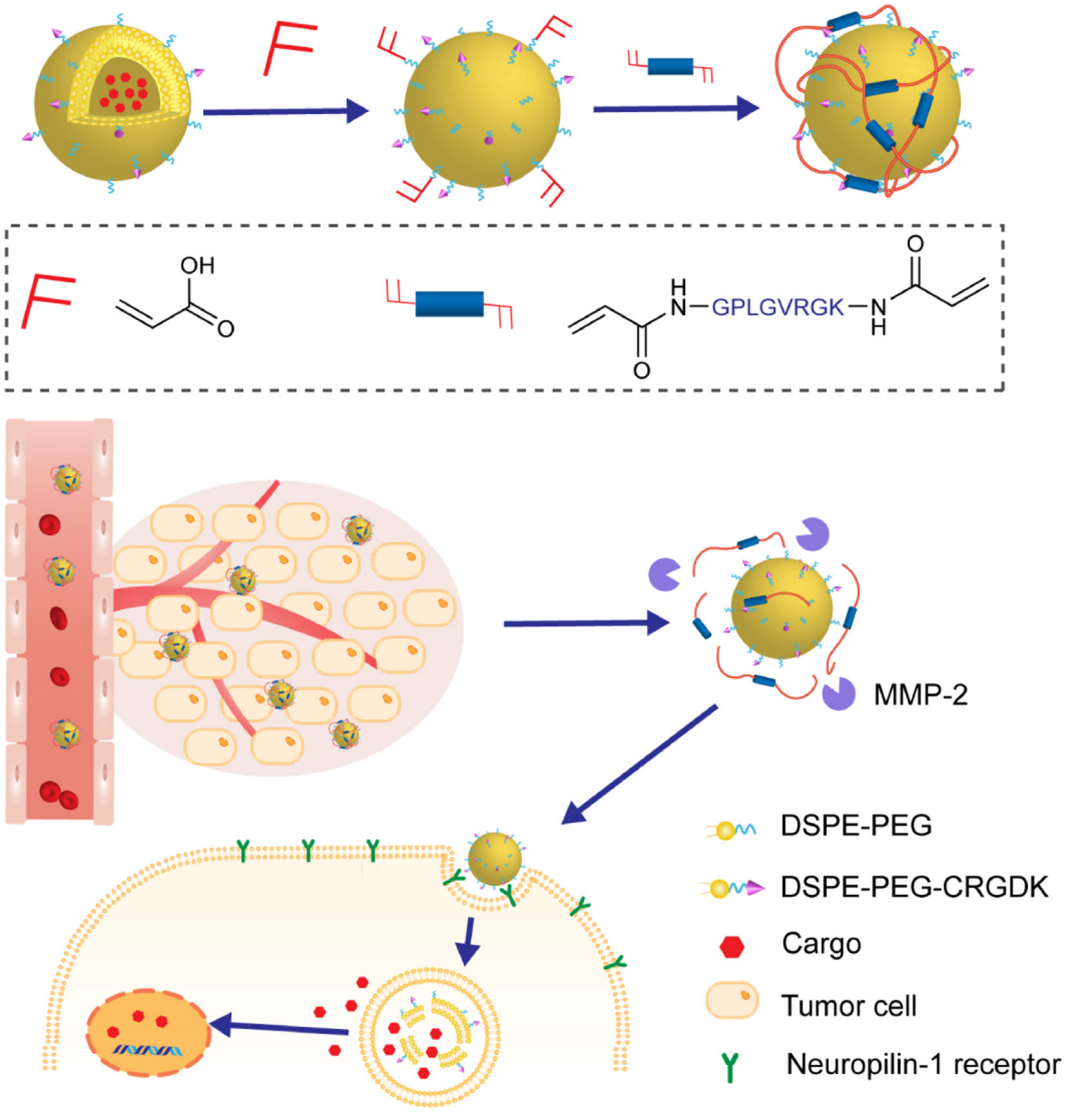
The nanoparticles were composed of nanovesicles as core and MMP-2 responsive polymeric network as crown. The crown disassembled in the tumor microenvironment, enhancing the penetration and internalization of nanovesicles.

Instead of being composed entirely of peptides, the crown can also be consisted of synthetic hydrophilic polymers cross-linked by enzyme-cleavable peptides. In this way, triggered-release of drugs could be achieved via partial degradation of polymeric crown. Chen et al. reported a delivery platform based on nanoparticles bearing synthetic PEGs as hydrophilic crown. It was further functionalized with MMP-2 responsive peptides GPLGVRGK and tumor-targeting ligand CRGDK peptides, as illustrated in Figure 6 (Liu et al., 2015). The presence of PEGs was supposed to extend the circulation time in blood and the installation of GPLGVRGK peptides was designed to provide additional prevention from undesired drug leakage and enzyme-triggering sites. The nanoparticles accumulated in the tumor location through the EPR effect, followed by the breakage of the crown in response to the over-expressed MMP-2 in the tumor microenvironment. With the navigation effect of the tumor-targeting ligand CRGDK peptide, enhanced deep-tissue penetration and cellular internalization were achieved, which significantly improved the therapeutic efficacy.

**Figure 7:**
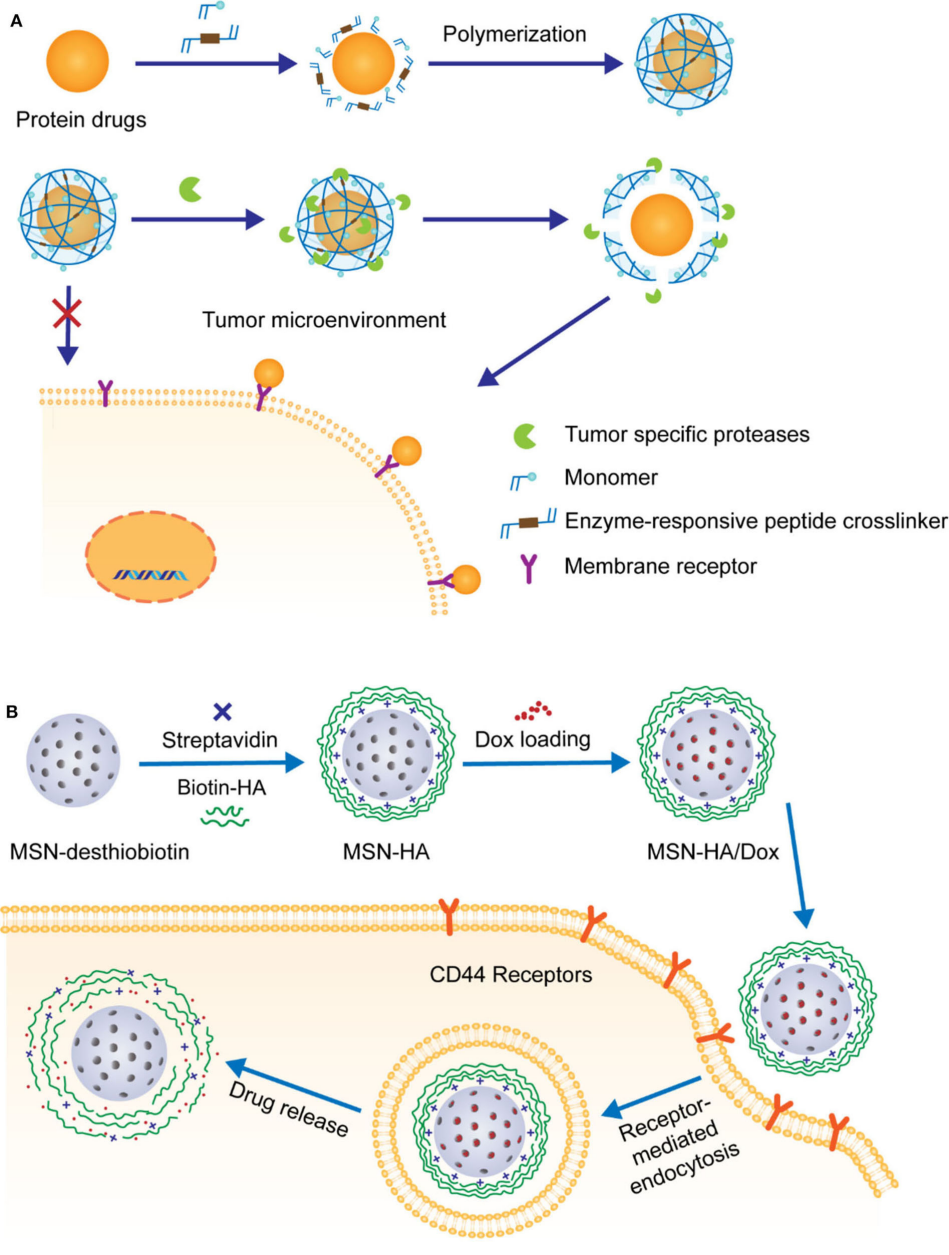
**(A)** Schematic illustration of MMP-responsive nanocarriers prepared by *in situ* polymerization and their applications for extracellular delivery of therapeutic peptides. **(B)** Schematic illustration of the decoration of Dox-loaded MSN with HA and HAase-triggered release of drugs.

Different from being active in diseased cells, extracellularly functional protein drugs with high safety and excellent specificity have recently emerged as important alternatives for clinical applications. However, like other protein-based drugs, the intrinsic fragility and susceptibility of these drugs to complex *in vivo* conditions make their clinical practice formidable. And thus, it is highly desirable to develop weakly cell-interacted, nanosized, and enzyme-responsive NPs. Lu and coworkers have developed a mild *in situ* polymerization process to construct nano delivery platforms of protein drugs with controllable enzyme-response capabilities, as shown in Figure 7A. In 2015, they reported the fabrication of nanocapsules with plasmin-responsive crown via *in situ* polymerization (Zhu et al., 2015). The monomers contained peptide linkages made from different enantiomers of amino acids. Spatiotemporal control of these nanocapsules in response to plasmin could be achieved by changing the chirality of peptide linkages in the crown. In their follow-up study, MMP-2-responsive peptides with highly hydrophilic zwitterionic phosphorylcholine were utilized to modify the crown of NPs (Li et al., 2019). By optimizing the filling rate of phosphorylcholine in crown, the interaction between NPs and cells could be effectively weakened and thus the undesired internalization by tumor cells and the loss of enzyme-recognizable peptides could be avoided. Recently, this platform was further improved to deliver monoclonal antibodies for brain tumor treatment (Han et al., 2019). In this case, the crown of nanocapsules was constructed via *in situ* polymerization of 2-methacryloyloxyethyl phosphorylcholine (MPC) and MMP-2-cleavable peptide crosslinker.

Recently, HA has been frequently reported due to its potential multiple roles in the development of anti-tumor therapy. First of all, similar as PEG, the high hydrophilicity, non-toxicity, and biodegradability of HA make it ideal for the coating of anti-tumor drug delivery NPs. In addition, it has been demonstrated that HA is of strong and specific targeting ability toward CD44, a transmembrane glycoprotein overexpressed on various tumor cells. Furthermore, HA is composed of enzyme-degradable N-acetylglucosamine and D-glucuronic acid disaccharide units, rendering it a good candidate for fabricating NPs with hyaluronidase (HAase)-responsive crown. For example, Zhang and coworkers prepared mesoporous silica nanoparticle (MSN)-based delivery vehicles coated with biotin-modified HA, achieving targeted delivery and controlled release of anti-tumor agent doxorubicin hydrochloride (Dox) in the tumor cells with overexpression of HAase, as shown in Figure 7B (Zhang M. Z. et al., 2016). The benefit of this enzyme-responsive strategy was approved by *in vitro* analyses, which showed that the simultaneous presence of biotin and HAase significantly facilitated the release of Dox.

**Figure 8:**
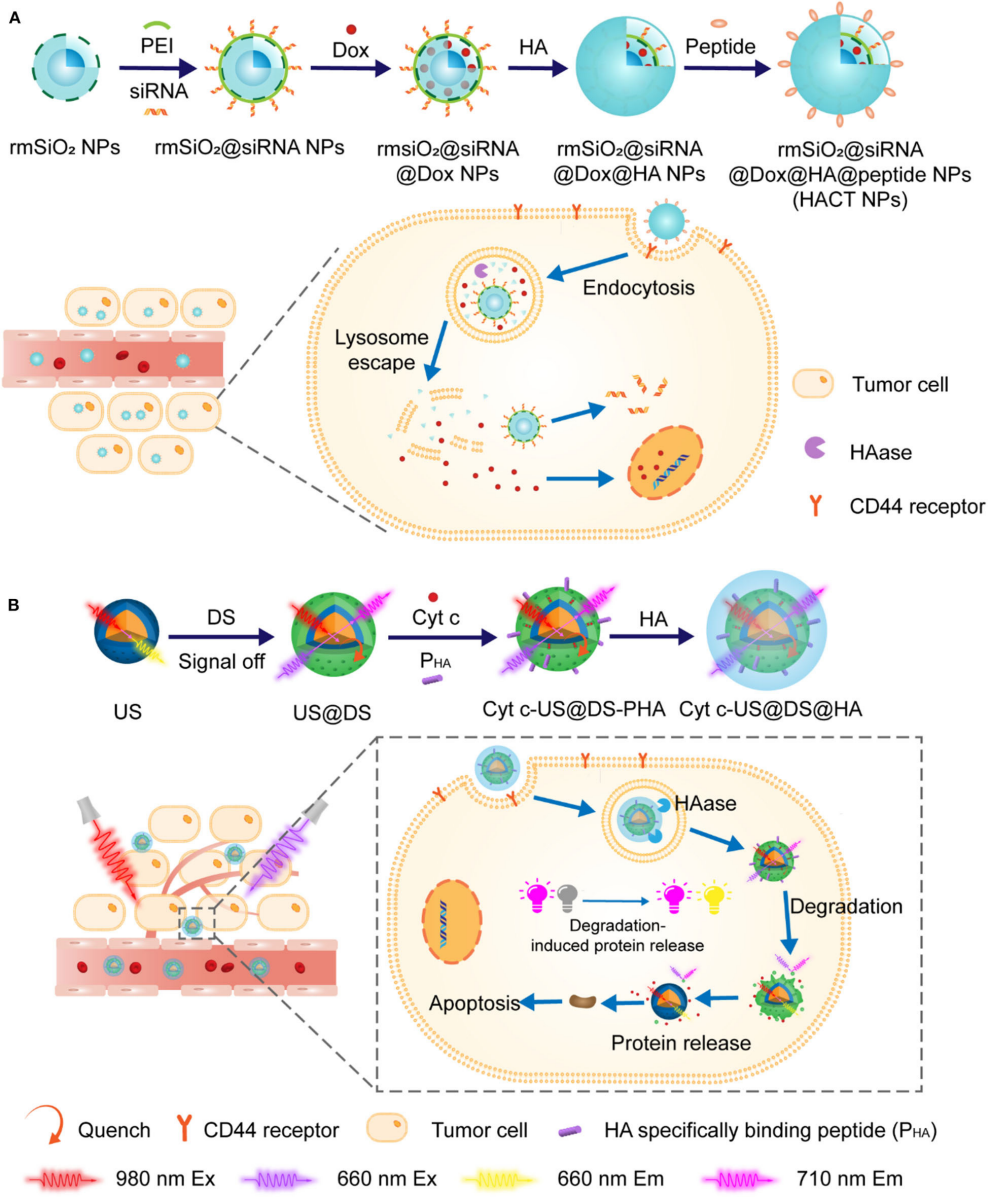
**(A)** Schematic illustration of the construction of HACT NPs through “layer-by-layer assembly” strategy. HA crown decorated with peptides allowed pinpointed delivery of siRNAs along with Dox. **(B)** Schematic strategy of using outer-frame-degradable nanovehicles with NIR dual luminescence to monitor the biological distribution of nanoparticles and the release of therapeutic proteins.

In another study by Min group, similar MSN with a hallow and rattle structure (rmSiO_2_ NPs) were utilized for the codelivery of siRNAs and Dox in an enzyme-responsive fashion (Ding et al., 2017). In order to effectively load both hydrophilic negatively charged siRNAs and hydrophobic Dox, a “layer-by-layer assembly” strategy was adapted for the fabrication of NPs with HA on the top surface. These NPs were further functionalized with breast tumor cell homing and penetrating-peptide PEGA-pVEC, as illustrated in Figure 8A. The resulting novel NPs with cascade targeting capabilities showed enhanced selective accumulation in tumor microenvironment and HAase-triggered controllable release of drugs in the targeted tumor cells. As more and more NPs with therapeutic proteins or peptides as payloads have been exploited for tumor treatment, it is urgent to fully reveal the mechanism and pharmacological efficacies in tumor therapy via real-time tracking of therapeutic proteins. For this reason, the same group artfully designed and prepared an outer-frame-degradable nanovehicle by coupling upconversion nanoparticles (UNCPs) with fluorophore-doped macroporous silica (DS). It was finally coated with enzyme-responsive HA crown, as illustrated in Figure 8B (Zhang et al., 2019). As expected, *in vitro* and *in vivo* evaluation demonstrated that both biodistribution of nanovehicles and the HAase-induced release of protein could be visually monitored at different NIR fluorescence channels. Interestingly, in addition to be a monitoring platform, this nanovehicles with cytochrome loaded also showed excellent anti-tumor therapeutic efficacy.

**Figure 9:**
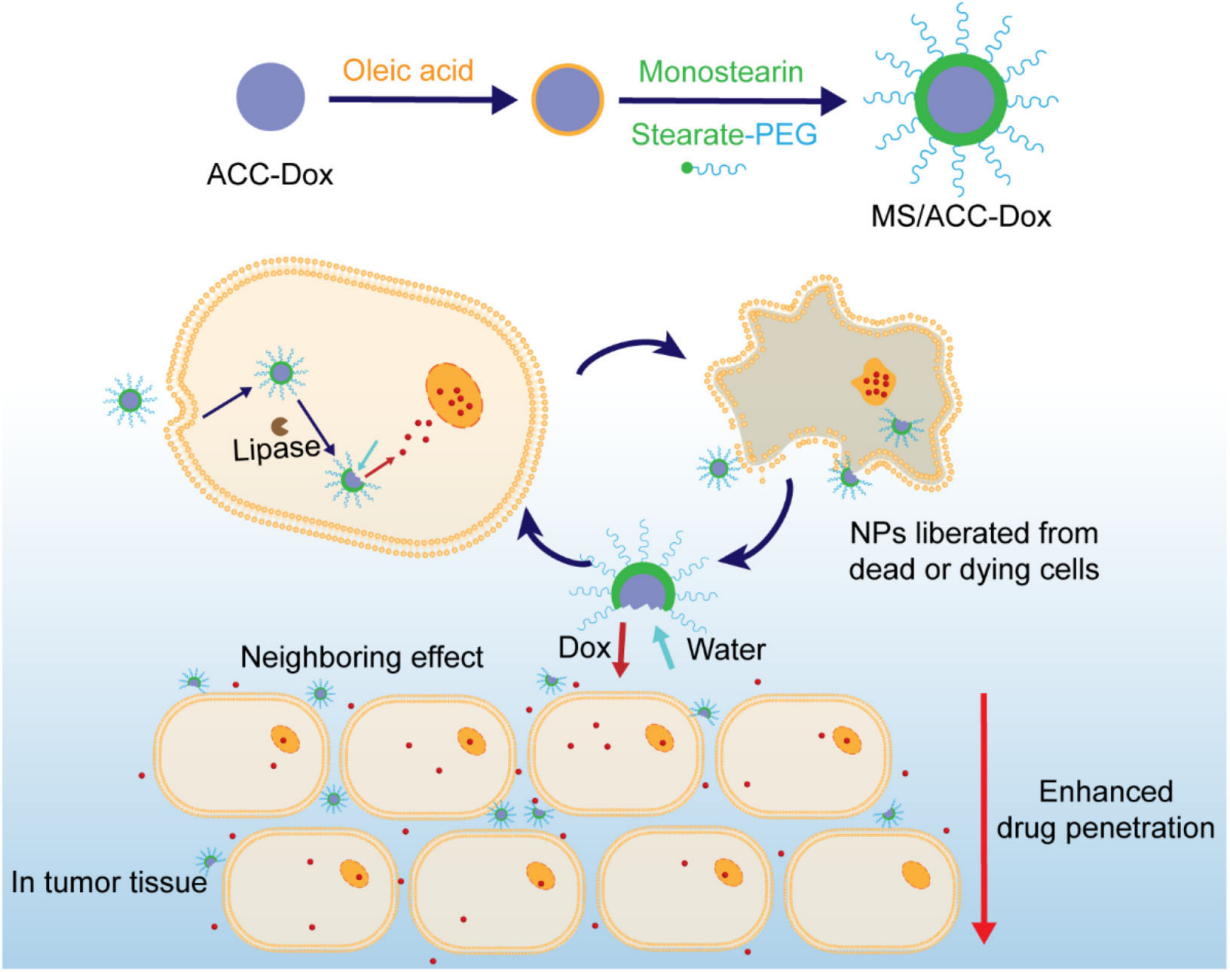
Schematic illustration of the decoration of water-sensitive and Dox-loaded ACC with lipase-responsive monostearin. Drugs were released under the trigger of lipase and water.

Quite similar to HAase-responsive HA/MSN nanodelivery systems discussed above, lipase-triggered monostearin/amorphous calcium carbonate (MS/ACC) NPs loaded with Dox have been reported by Wang et al. (2018), as shown in Figure 9. In this case, a new kind of enzyme-responsive combination MS/lipase has been introduced, along with protein or peptide/protease and HA/HAase. It is noteworthy that the MS/ACC NPs exhibited additional water-sensitivity due to the high degradability of ACC in aqueous media, which proved to be crucial to induce a neighboring effect and enhance drug penetration. With Dox loaded, MS/ACC-Dox nanoparticles showed a significant effect on inhibiting tumor growth on SKOV3 xenografted nude mice.

### Nanoparticles With Enzyme-Responsive Linker

Normally, cleavable linkers are an essential component of NPs. They can be utilized to attach drugs to the hydrophobic core, or connect hydrophobic core with hydrophilic crown, or modify the hydrophilic surface with targeting ligands. Ideal cleavable linkers should ensure the auxiliary of NPs remains attached during circulation but cleaves rapidly after NPs reach the targeting action sites. It is desirable if the cleavable-linker can be endowed with enzyme-responsive capability (Bohme and Beck-Sickinger, 2015). In this context, peptides with specific protease responsibility are considered as the most common candidates for fabricating NPs with enzyme-responsive linkers.

**Figure 10:**
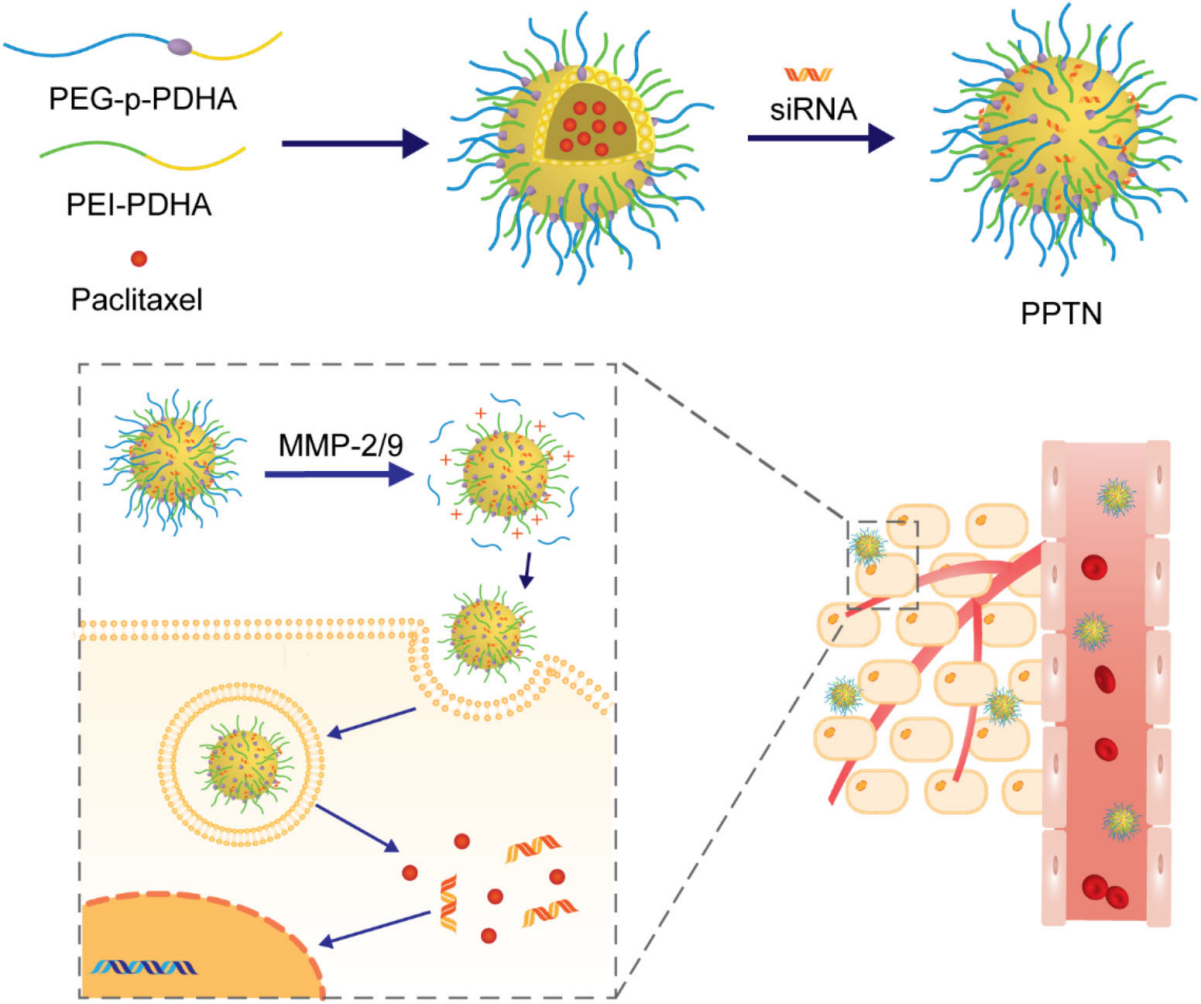
Schematic illustration of construction of the siRNA and paclitaxel loaded micelle PPTN and subsequent activation in response to MMP-2/9.

**Figure 11:**
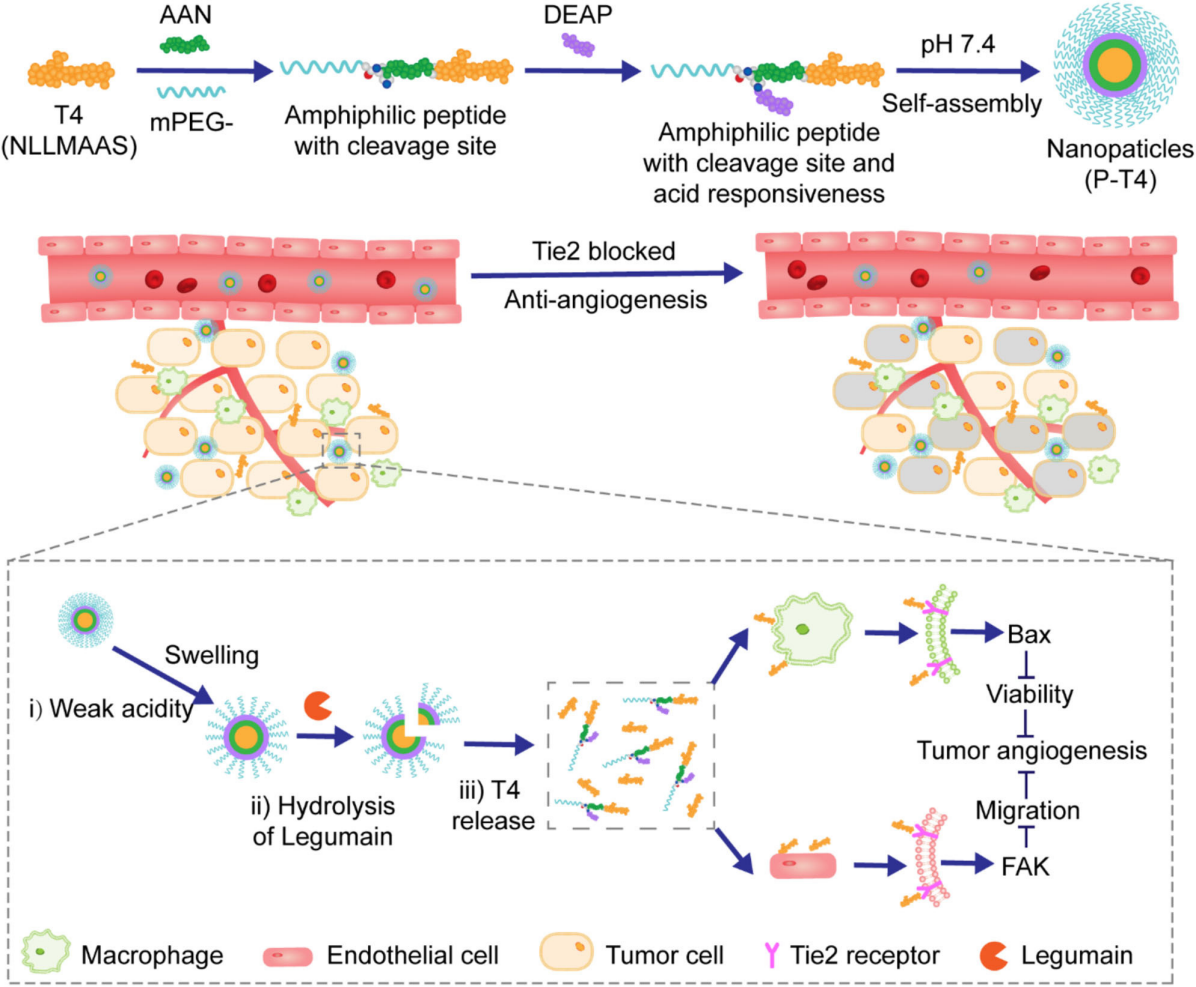
Schematic illustration of the fabrication of the nanoparticles and the proposed mechanism of pH and legumain-responsive release of therapeutic peptides T4.

MMPs are among the most studied proteases for applications in antitumor drug delivery systems and some cases with MMP-degradable peptides as linkers have been reported. For example, Yin and coworkers designed and prepared a nanoparticle with highly hydrophilic PEG as cationic charge shielding surface. It was attached to active agents-loaded core via an MMP-degradable peptide linker Pro-Leu-Gly-Leu-Ala-Gly (PLGLAG), as illustrated in Figure 10 (Tang et al., 2016). In this study, it was demonstrated that these long circulating NPs could be passively localized in the tumor tissues via the EPR effect. In the presence of PLGLAG-sensitive MMP-2/9, the PEG layer fell off and the resulting exposure of positive charges promoted the uptake of NPs by the tumor cells. In another study, NPs fabricated in a similar way was reported by Zhang et al. (2019). In this approach, fragile hydrophobic therapeutic small peptides T4 (NLLMAAS) were rationally modified with PEG via an enzyme-responsive linker of amino acid sequence AAN, as illustrated in Figure 11. The linker is cleavable in tumor cells and tumor-related microenvironment with overexpression of cysteine protease legumain.

**Figure 12:**
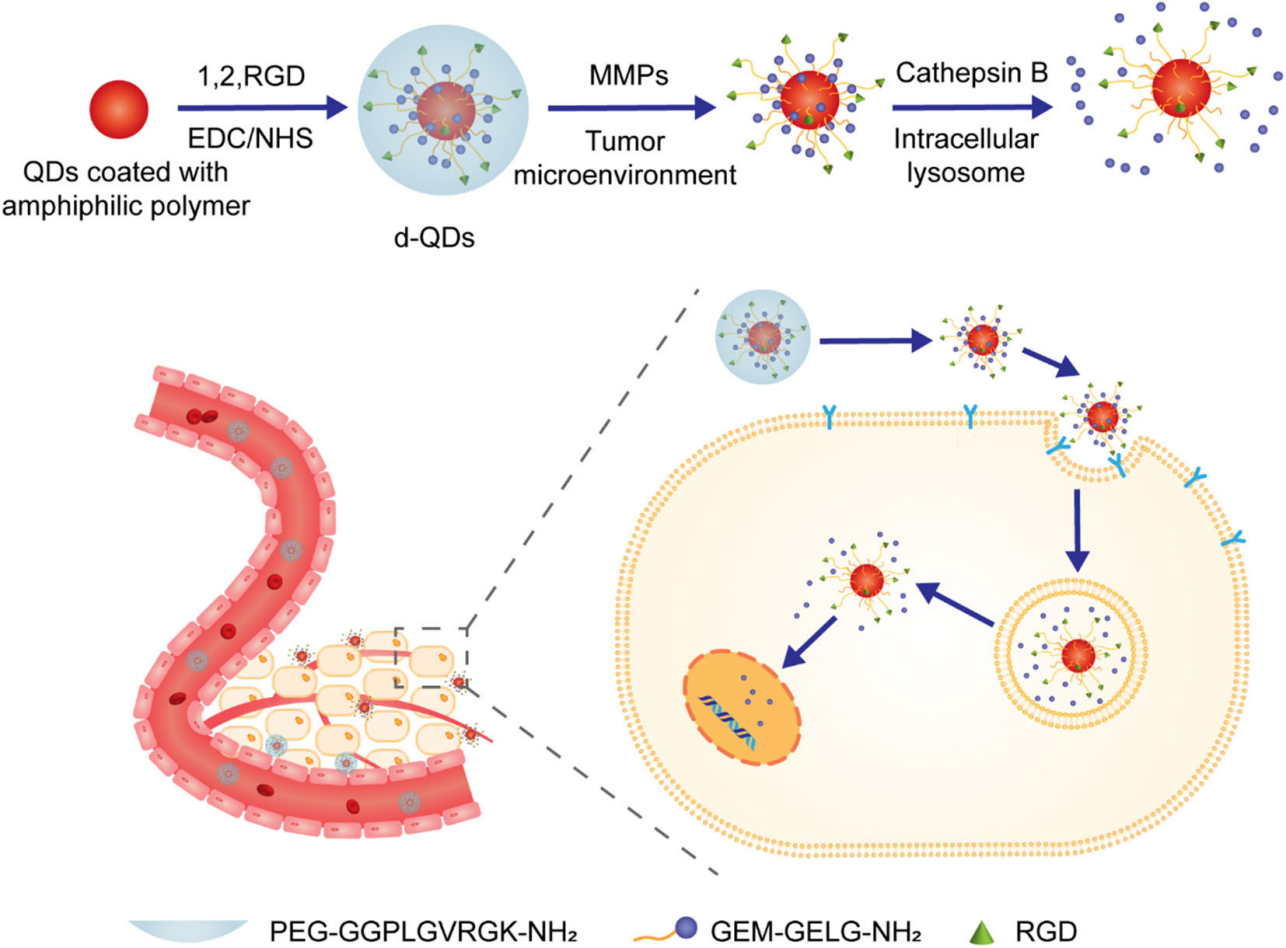
Schematic illustration of preparation of QDs-based nanoparticles and their enzyme-triggered behavior.

Like silica-and calcium carbonate-based nanoparticles, inorganic quantum dots (QDs) have also been explored as platforms for drug delivery, for which surface modification with hydrophilic motifs are deemed essential. Jin et al. reported CdSe/ZnS QDs-based NPs for the delivery of anti-pancreatic cancer therapeutic gemcitabine (GEM) (Han et al., 2017). In this study, commonly used PEG was utilized to decorate the surface of QDs through the linkage of MMP-9 substrate peptide GGPLGVRGK. In addition, a second cathepsin B-sensitive peptide linker GFLG was installed between QDs and GEM, as illustrated in Figure 12. It is well demonstrated that MMP-9 is overexpressed in the pancreatic tumor microenvironment while cathepsin B is up-regulated in the pancreatic tumor cells. This novel NPs delivery system with two sequential enzyme-responsive linkers showed enhanced accumulation of the activated form of GEM, reduced side effects, and superior tumor suppression ability.

**Figure 13:**
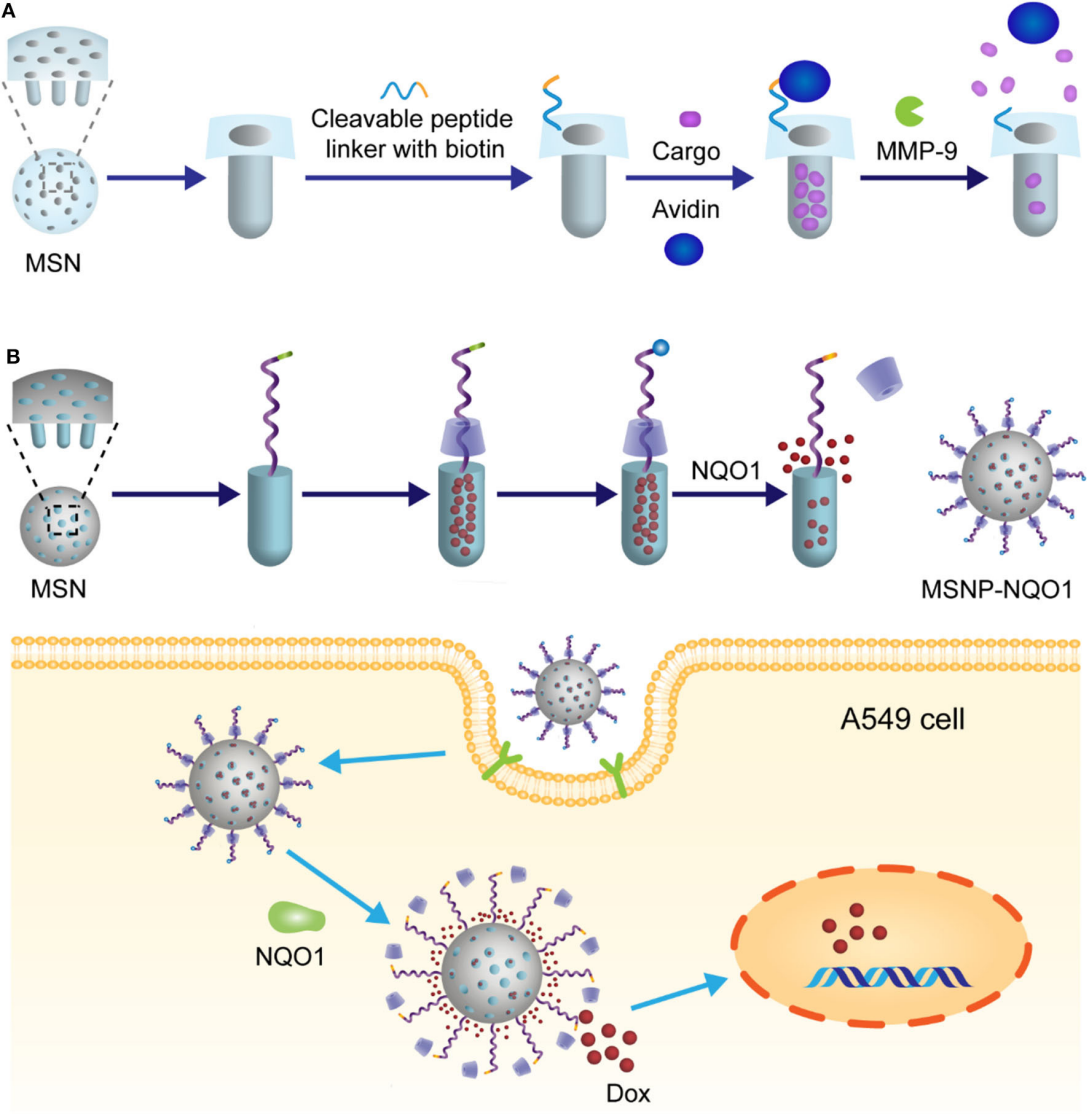
**(A)** Schematic illustration of MMP-9 mediated release of MSN nanoparticles. In this system, controlled release of the drug was achieved through avidin linker which was used to cover the surface with MMP-9 substrate peptides. **(B)** Schematic illustration of MSN nanoparticles and their NQO1 triggered release mechanism.

MSN-based drug delivery systems have also been successfully modified with enzyme-responsive peptide linkers. van Rijt et al. developed a novel approach for controllable release of anti-tumor therapeutics mediated by MMP-9 (van Rijt et al., 2015). Firstly, the external surface of the MSNs was coated with biotins via heptapeptide linkers, which bear MMP-9-recognizable and cleavable sequence RSWMGLP. After loading therapeutics, the outer surface of the NPs was readily covered with hydrophilic avidins which are of high affinity for biotins, as shown in Figure 13A. The resulting well-armed NPs induced significant apoptosis of tumor cells in lung tumor regions of mice, while showing non-toxicity in tumor-free tissues or in healthy mice.

Another interesting example involving MSNs was presented by Gayam et al. (2016). In this study, a new kind of enzyme-responsive combination quinone/quinone oxidoreductase 1 (NQO1) was introduced and utilized for the design of enzyme-triggered drug delivery system, because overexpression of NQO1 in several human tumor cells has been demonstrated. Strictly speaking, it is not the enzyme-stimuli cleavage of linker that directly leads to the release of drugs. In this case, as illustrated in Figure 13B, the Dox loaded in MSNs was capped by an α-cyclodextrin with a stalk going through. The end of the stalk was functionalized with a benzoquinone, which acts both as a stopper to lock the α-cyclodextrin and a reactive site toward NQO1/NADH. Interestingly, in the presence of NQO1, the stopper benzoquinone was reduced to hydroquinone, followed by self-cleavage from the stalk. As a result, the gatekeeper α-cyclodextrin was freed and thus DOX was released. This delicate drug delivery system successfully avoided the premature release of drugs.

### Nanoparticles With Enzyme-Responsive Ligand

In order to achieve precise delivery of anti-tumor drugs and provide with personalized medicine due to the high heterogeneity degree of tumor cells, the strategy to arm the delivery vehicle with a targeting ligand has been accepted and implemented. The design and selection of targeting ligands mostly depend on the receptors overexpressed in diseased tissues. In current clinical studies, a wide variety of targeting ligands have emerged. Peptides and HA play important roles in the development of targeting ligands with enzyme-responsive ability.

**Figure 14:**
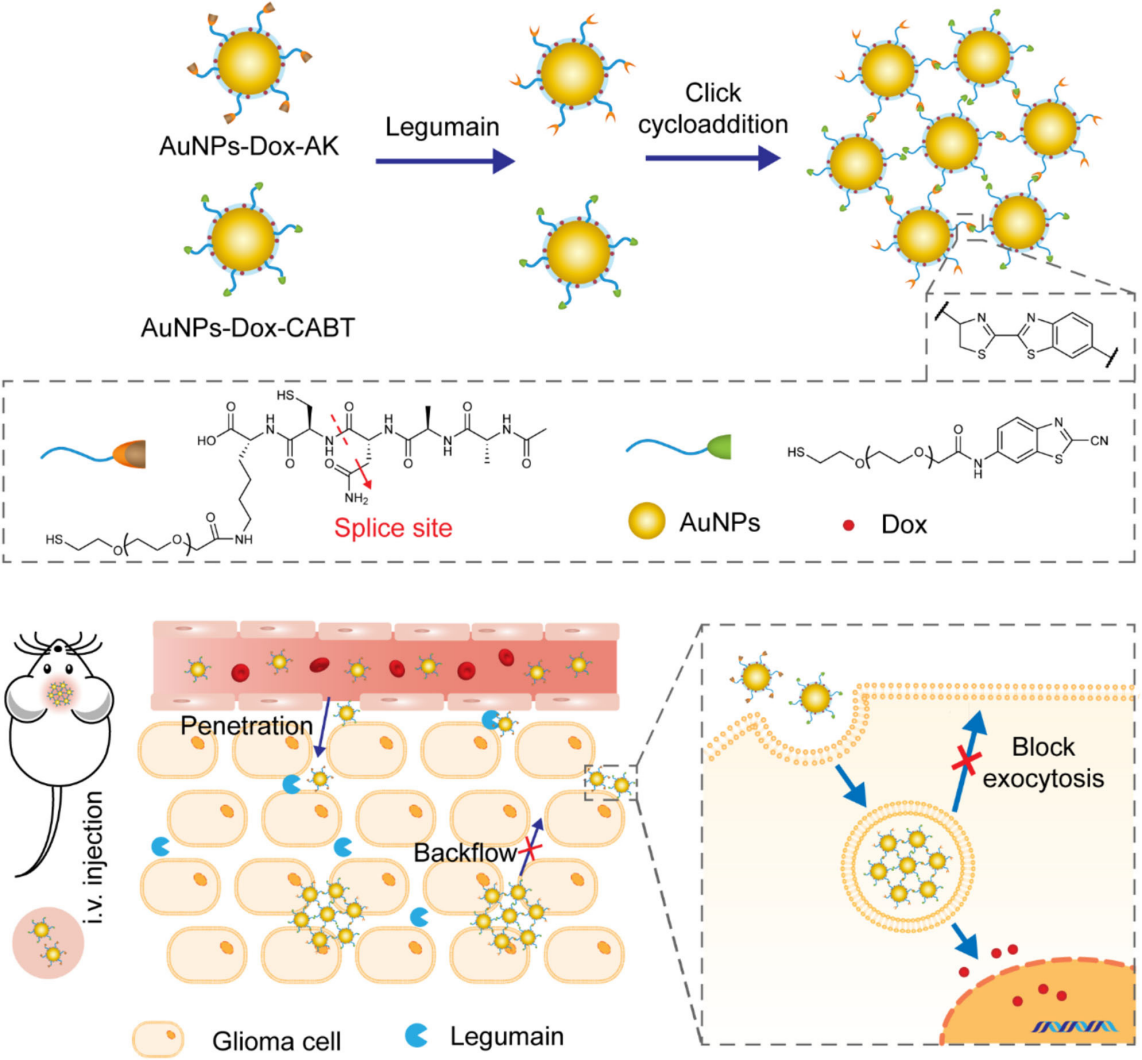
Schematic diagram showing the design of nanoplatform which aggregated under the action of legumain and achieved good retention in brain tumors.

Non-specific interactions of NPs with healthy tissues can be largely avoided by using enzyme-responsive materials as targeting ligands, as ligand-guided dynamic activities will occur only when NPs are exposed to specific tumor microenvironments. The aggregation guided by enzyme-responsive ligand has great importance in facilitating the penetration of NPs through the blood-brain barrier and enhancing retention of NPs in brain tumors. For example, Gao et al. developed gold nanoparticles (AuNPs) capable of aggregating in brain tumor cells with overexpression of legumain (Ruan et al., 2016). This nanoplatform was comprised of two kinds of AuNPs with different ligands, as illustrated in Figure 14. One ligand was designed to be legumain-specific substrate which could expose its 1,2-thiolamino groups via legumain-catalyzed hydrolysis. The other one bearing cyano groups would readily react with the 1,2-thiolamino groups via click cycloaddition, leading to the formation of AuNPs aggregates. As a result, the newly formed AuNPs with expanded size could effectively block nanoparticle exocytosis and minimize nanoparticle backflow to the bloodstream.

**Figure 15:**
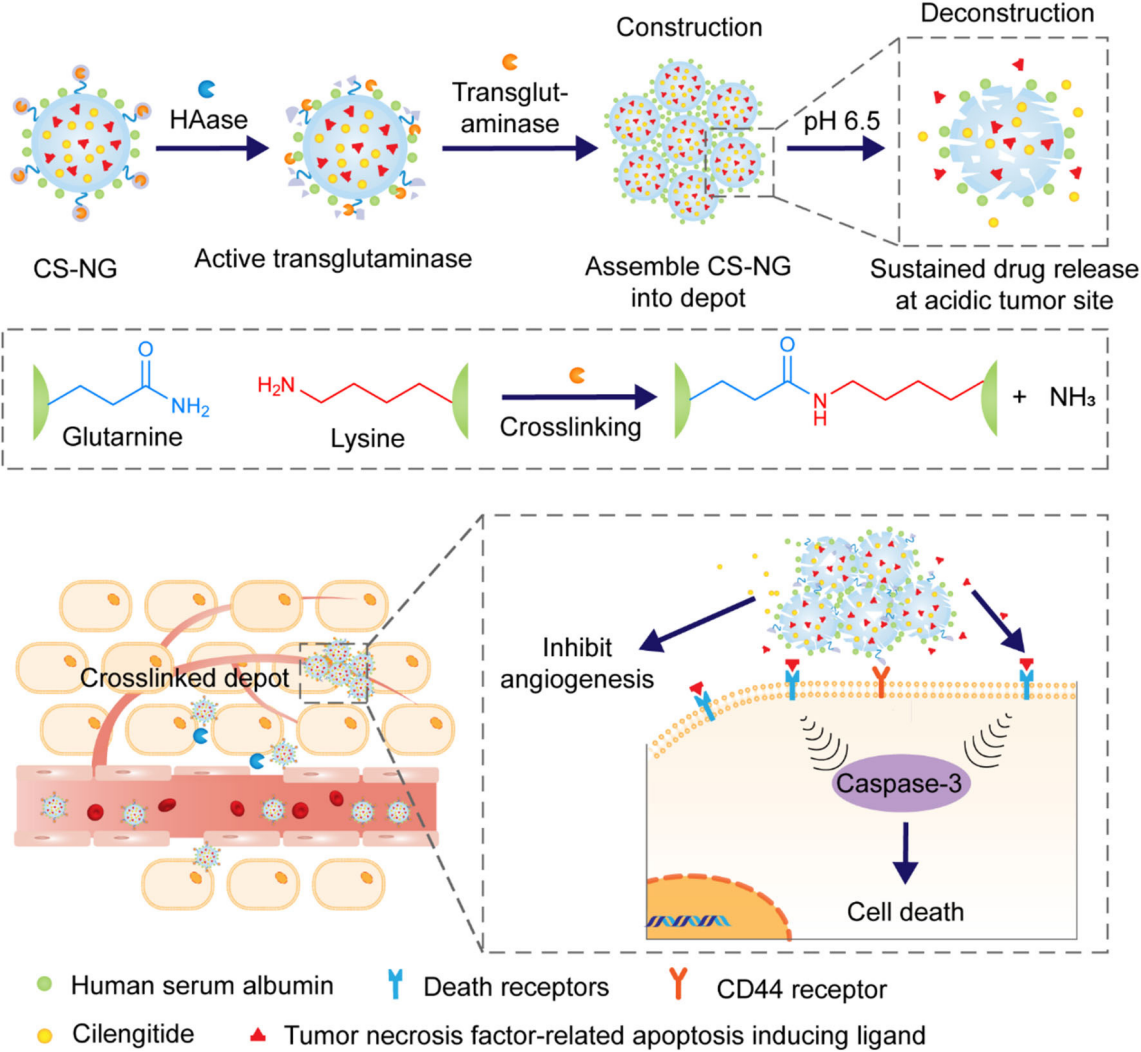
Schematic diagram depicting the tumor microenvironment-mediated construction and deconstruction of extracellular drug depots for sustained drug release.

Not only for the delivery of intracellular active drugs, this strategy of enzyme-induced NPs aggregation is also applicable for the delivery and continuous release of extracellular active proteins/peptides in tumor tissues since the aggregated NPs as depots are not easy to be internalized by the cells. Gu group designed a nanoplatform (CS-NG) which could assemble into microsized extracellular depots via transglutaminase-catalyzed cross-linking of human serum albumin (Hu et al., 2016), as showed in Figure 15. HA matrix loaded with transglutaminase as an enzyme-responsive ligand and human serum albumin were collectively coated on the surface of glycerol dimethacrylate-based core preloaded with therapeutics. After NPs accumulated in tumor tissues with overexpression of HAase, transglutaminase was released from HA matrix, which further triggered the cross-linking of HA and formed microscale “drug-delivery depots” as reservoir to continuously release therapeutics.

## Conclusions

Aberrantly high expression of tumor-associated enzymes is a feature of the tumor microenvironment, which can be utilized to design anti-tumor drug delivery systems based on nanoparticles with enzyme-response capability. In this review, four types of enzyme-responsive NPs were introduced depending on the different components of NPs on which an enzyme takes action. Different effects, such as better tissue or membrane penetration, reduced toxicity, extended circulation time, improved accumulation, and controllable release of active therapeutics, can be achieved by fabricating NPs with enzyme-responsive sites. NPs with enzyme-responsive crown are the most common carriers for antitumor drug delivery because of their relatively simple structure, easy preparation, and short response time. More attention should be paid to these NPs due to their great potential for clinical applications in the future. Among the enzymes explored and evaluated, proteases with versatile response abilities toward different enzyme-sensitive components of NPs have been frequently investigated. Besides as therapy for tumor treatment, enzyme-responsive NPs have also been used for tumor monitoring and localization as they can target diseased tissues and accumulate in tumor microenvironments with desired sensitivity. In addition, it has been well demonstrated that codelivery of multiple payloads by these enzyme responsive systems is quite achievable. For all of these reasons, the exploration and clinical applications of the enzyme-responsive NPs applications will undergo considerable expansion.

Although great progress has been made in the design and application of enzyme-responsive nanoparticles, there are still many challenges that need to be addressed. First of all, considering the high complexity of tumor microenvironment, there is a tremendous variety of enzyme activity dysregulation in different cancers and even the same cancer at different stages of progress. It is really difficult to build a general enzyme-responsive nano delivery platform for anti-tumor therapeutics. And due to the high heterogeneity degree of cancer, even more difficulty could be envisioned.

Secondly, targeted and controlled release of drugs from enzyme-responsive NPs relies on the high reactivity of enzymes for their substrates with exceptional selectivity. This exclusive one-to-one relationship between enzyme-responsive NPs and tumor microenvironment with overexpression of the exact enzyme is not as solid as assumed. Take MMPs for example, this family of over 20 proteinases have similar catalytic mechanisms and thus substrate preferences. NPs modified with short peptide substrates could be sensitive to various tumor microenvironments. Therefore, rational chemistry design of enzyme-specific substrates for fabricating NPs with precise enzyme-response ability is required.

Thirdly, compared with improved anti-tumor efficacy of enzyme-responsive NPs clearly illustrated *in vitro* investigations, there is still limited information about the underlying action mechanism of enzyme-responsive NPs *in vivo*. In addition, although positive results have been obtained in animal models, therapy based on enzyme-responsive NPs is still far away from being available for clinical use. Rational animal models should be developed and closer correlation between developed xenotransplantation models and clinical trials should be established.

Finally, a wide variety of enzyme-responsive NPs with sophisticated structures have been developed and some of them are undergoing clinical trials. Concern about their biosafety should be the key issue. Furthermore, due to their high structural complexity and multiple functionalities, enzyme-responsive NPs in development, especially those with high potential in clinical applications, might meet standards of homogeneity and the corresponding formulation techniques should be reproducible.

In summary, enzyme-responsive NPs hold great potential for more precise diagnosis and more effective treatment of cancers. There is a long way to go before we, with the assistance of enzyme-responsive NPs, eventually find a cure for cancer.

## Author Contributions

QS conceived the review topic and modified the manuscript. ML wrote the manuscript and arranged all the figures. All authors contributed to the final manuscript.

## Conflict of Interest

The authors declare that the research was conducted in the absence of any commercial or financial relationships that could be construed as a potential conflict of interest.
